# Executive function is inversely correlated with physical function: the cognitive profile of adult Spinal Muscular Atrophy (SMA)

**DOI:** 10.1186/s13023-020-01661-9

**Published:** 2021-01-06

**Authors:** Lucas Mix, Olivia Schreiber-Katz, Claudia D. Wurster, Zeljko Uzelac, Sophia Platen, Christina Gipperich, Gresa Ranxha, Gary Wieselmann, Alma Osmanovic, Albert C. Ludolph, Susanne Petri, Dorothée Lulé

**Affiliations:** 1 Department of Neurology, Ulm University, Oberer Eselsberg 45, 89081 Ulm, Germany; 2 Department of Neurology, Hannover Medical School, Hannover, Germany; 3 German Center for Neurodegenerative Diseases Ulm, Ulm, Germany

**Keywords:** Spinal muscular atrophy, SMA, Cognition, Executive function, Social cognition, Cognitive adaptation, Edinburgh cognitive and behavioural ALS screen (ECAS), Reading the mind in the eyes test, Hammersmith functional motor scale expanded (HFMSE)

## Abstract

**Background:**

Spinal muscular atrophy (SMA) issues from mutations in the *survival of motor neuron (SMN) 1* gene. Loss or reduction of the SMN protein results in progressive muscle weakness. Whether this protein deficiency also affects cortical function remains unclear. While no data on adult patients exists so far, prior studies in children with SMA indicate cognitive abilities equal or even superior to healthy controls. This may suggest a possible compensatory—neuropsychological and interactional—process. The goal of this study was to assess the cognitive profile of adult patients with SMA, with a special focus on social cognition as a potential candidate for enhanced cognitive function through compensatory processes.

**Methods:**

In a cross-sectional design, N = 31 adult SMA patients (types II and III) were assessed for language, verbal fluency, memory, visuospatial abilities and executive function with the Edinburgh Cognitive and Behavioural ALS Screen and for social cognition with the Reading the Mind in the Eyes Test. Physical function was evaluated using the Hammersmith Functional Motor Scale Expanded. N = 19 neurologically healthy controls were matched with patients for age, sex and years of education.

**Results:**

In none of the abovementioned cognitive domains significant differences between SMA patients and controls were found. Among patients, no differences between type II SMA and type III SMA were detected for any domain. However, a trend towards better social cognition in patients with type II SMA, compared to those with type III SMA was observed. Furthermore, a significant inverse correlation of physical function and executive function was detected: lower motor function was associated with a better executive function.

**Conclusions:**

This study shows cognitive abilities in adult SMA in the normal range for all assessed domains. Thus, reduction of SMN protein has no obvious negative impact on cognitive function. Executive functions are identified as the only cognitive domain correlated with disease severity. Therefore, executive functions may play a role in the adaptation to physical restrictions in SMA, making them a promising target for future research.

## Background

Spinal muscular atrophy (SMA) is a rare neuromuscular disease with an incidence between 6.3 and 26.7 per 100,000 and a prevalence between 1 and 2 per 100,000 in Europe [1, 2]. It causes a progressive, proximally accentuated and symmetric weakness and atrophy of striated muscles with a very variable disease course. While patients with type I SMA have a disease onset in the first six months of life and mostly die in infancy, patients with SMA type II have an onset before month 18, learn to sit but never to walk and reach adolescence or even adulthood. SMA type III has an onset after month 18 and patients learn to walk and have a near normal life expectancy, whereas type IV SMA is a mild adult-onset subtype [3, 4].

With the recent development of new therapeutic agents like nusinersen, risdiplam or onasemnogene abeparvovec (Zolgensma^®^), the history of physical decline may change dramatically in SMA in the future, making it an interesting subject to research [5]. However, cognition in patients with SMA remains a widely unexplored field but for very few studies. On the one hand, it is known that physical interaction with the environment—an ability severely impaired in SMA—is important for normal cognitive development [6]. On the other hand, there are other inherited neuromuscular diseases like Duchenne Muscular Dystrophy (DMD) and valosin-containing protein (VCP)/p97-associated phenotypes (inclusion body myopathy (IBM), Paget's disease of the bone (PDB), frontotemporal dementia (FTD) and amyotrophic lateral sclerosis (ALS)), where the molecular cause for muscle degeneration also impacts cognition [7, 8]. Here, it shall be noted that the survival of motor neuron (SMN) protein, which is affected in SMA, is thought to play a developmental role in different regions of the forebrain [9]. Taking these aspects into consideration, one might assume that cognitive function in SMA may be at stake. However, literature suggests the contrary. In 1967 Dubowitz postulated normal intelligence in children with SMA and emphasized their “keen interest in their surroundings, their observational abilities and their mental acuity” [10]. An early study from 1987 found no difference in cognitive abilities between children with SMA and children with DMD [11]. However, numerous subsequent studies consistently found cognitive abilities superior to those found in DMD or typically developing children, which suspects no negative impact of SMN protein reduction on the development of cognitive function [12–14]. Moreover, research on younger children with type II SMA showed a richer vocabulary and earlier grammar development [15], as well as spatial cognition and spatial language superior to healthy controls [16, 17]. Further research found normal intelligence in children but enhanced intelligence in adolescents with SMA, which rather suggests a positive impact on cognitive development [18]. Researchers explain this enhanced performance either on an interactional level by assuming enhanced reliance on caregivers and thus an increased need for understanding social encounter [14], or on a neuropsychological level with the reallocation of cognitive resources otherwise used for sensorimotor development [19]. All these data apply to children or adolescents with SMA, while data for adult patients does not exist. Thus, it is unclear, if the suggested enhanced cognitive performance, found in adolescents with SMA, is preserved or even increased in adulthood. This study aimed at providing first evidence on the cognitive profile of adult patients with SMA. A special focus of this study laid on social cognition and here to assess whether the described “keen interest in their surroundings” [10] may be reflected in an especially well-developed social cognition in adulthood.

## Results

**Fig. 1:**
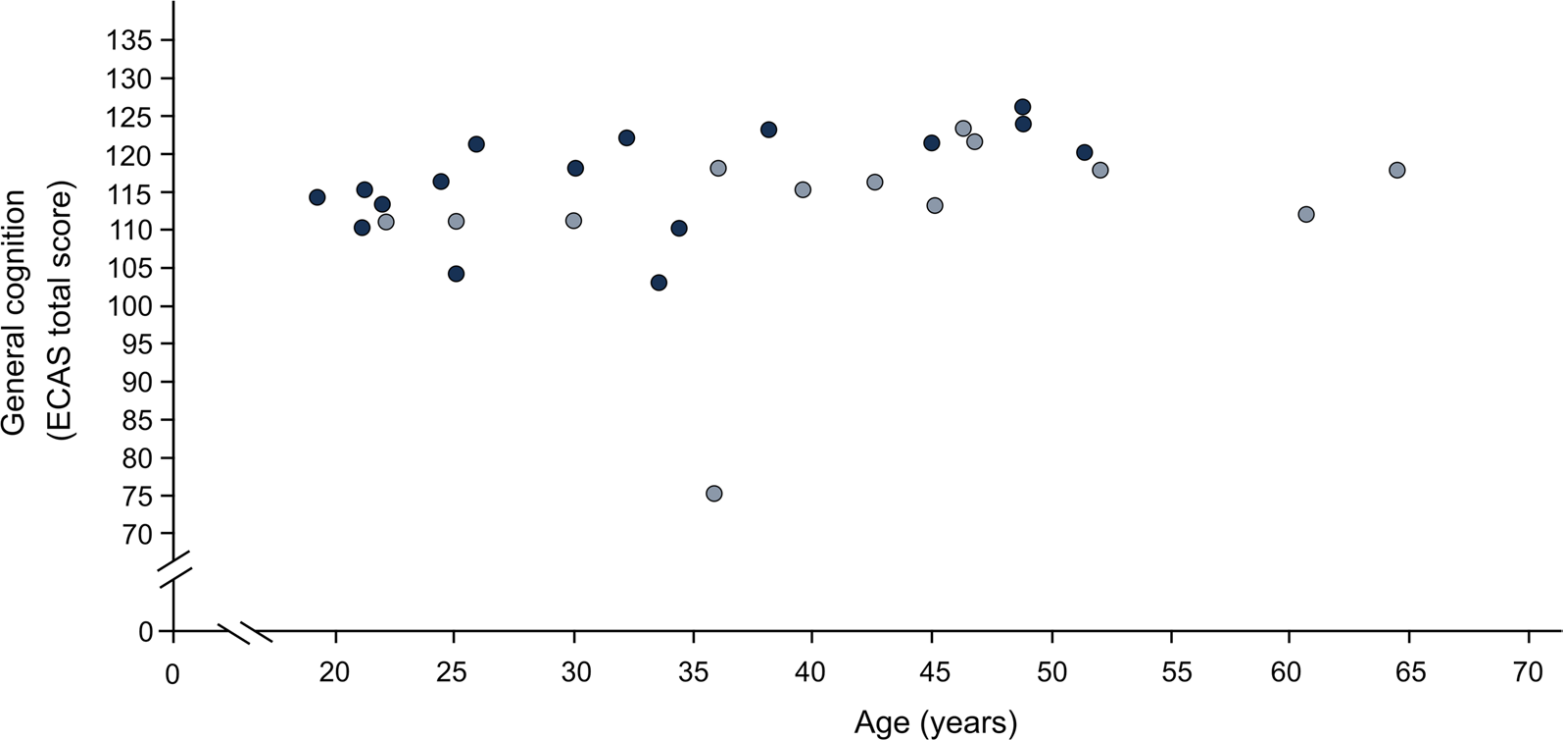
Association between general cognition (ECAS total score) and age in adult patients with SMA (N = 29). Dark blue = SMA type II, light blue = SMA type III; ECAS = Edinburgh Cognitive and Behavioural ALS (amyotrophic lateral sclerosis) Screen, total score (score 0 to 136); the data show a significant correlation between general cognitive skills (measured with ECAS total score) and age (τ = 0.30, *p* = 0.023)

**Fig. 2:**
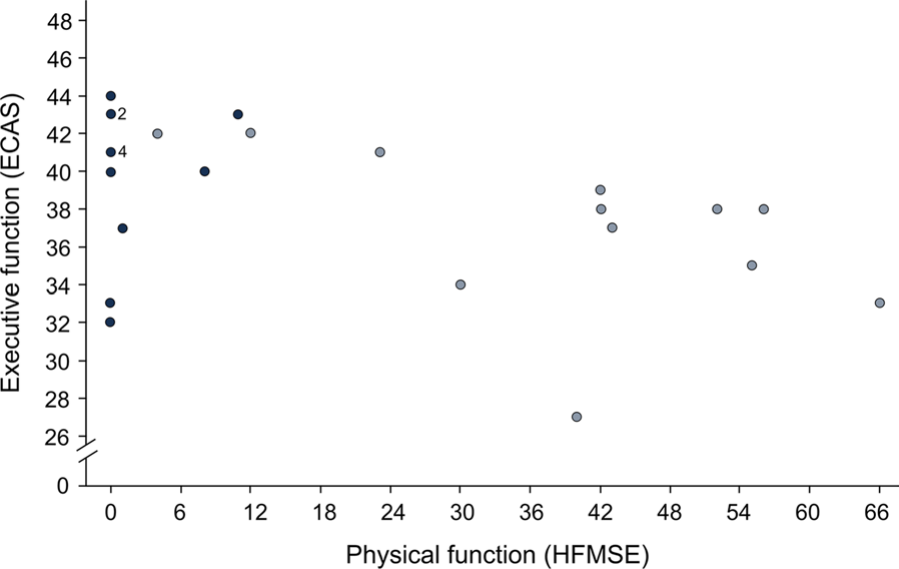
Association between executive function (ECAS domain) and motor function (HFMSE) in adult patients with SMA (N = 25). Dark blue = SMA type II, light blue = SMA type III ECAS = Edinburgh Cognitive and Behavioural ALS (amyotrophic lateral sclerosis) Screen, domain for “executive function” (score 0–48); HFMSE = Hammersmith Functional Motor Scale Expanded measures physical function on a scale from 0 to 66; the data point labelled “2”, represents two patients, the one labelled “4”, represents four patients; the data show a significant inverse correlation of executive and physical function (τ = − 0.36, *p* = 0.018)

**Fig. 3:**
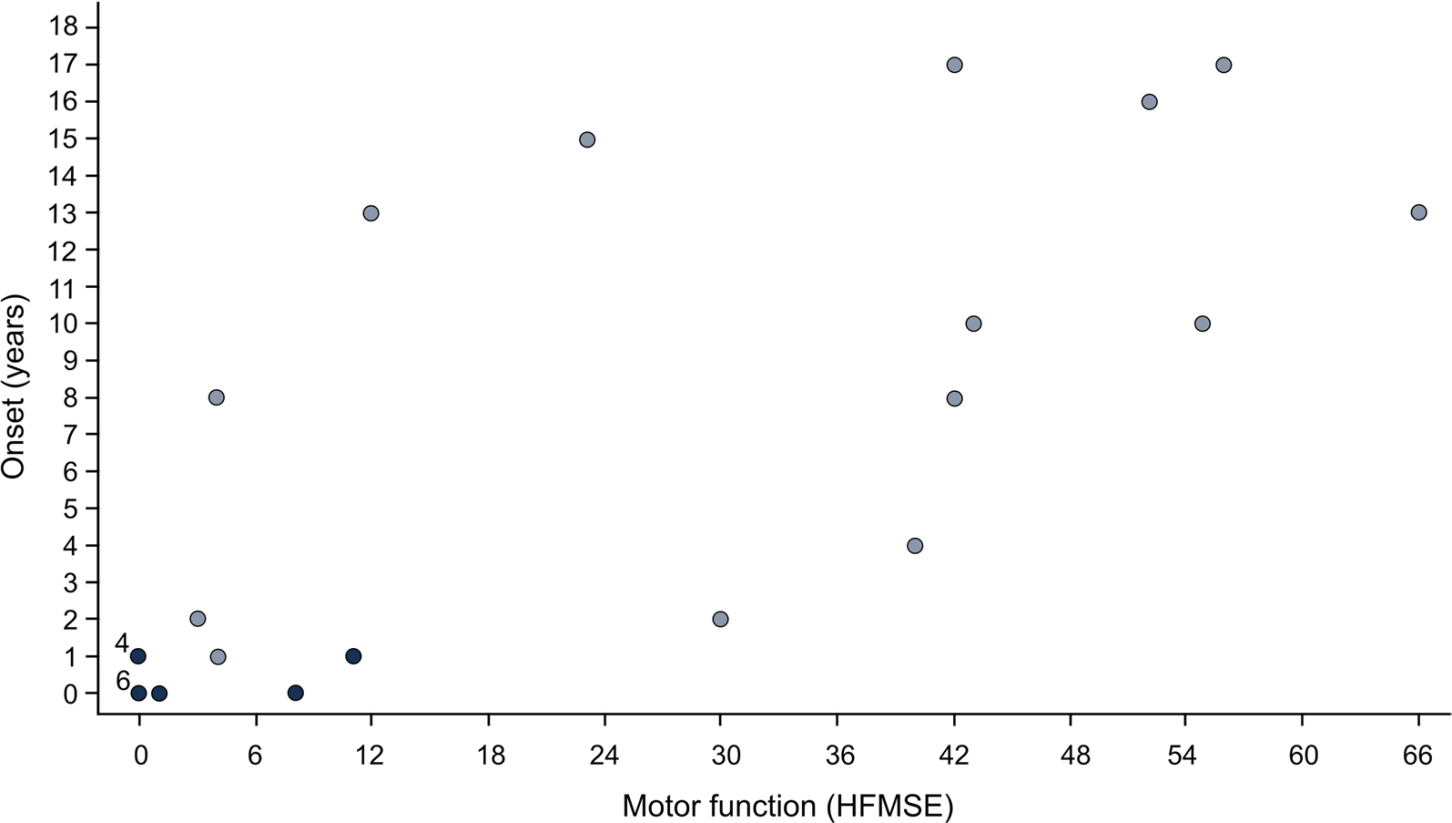
Association between disease onset and motor function (HFMSE) in adult patients with SMA (N = 27). Dark blue = SMA type II, light blue = SMA type III; HFMSE = Hammersmith Functional Motor Scale Expanded measures physical function on a scale from 0 to 66; the data points labelled “4” and “6”, four and six patients respectively; the data show a significant correlation of age at disease onset and physical function (τ = 0.69, *p* < 0.001)

**Fig. 4:**
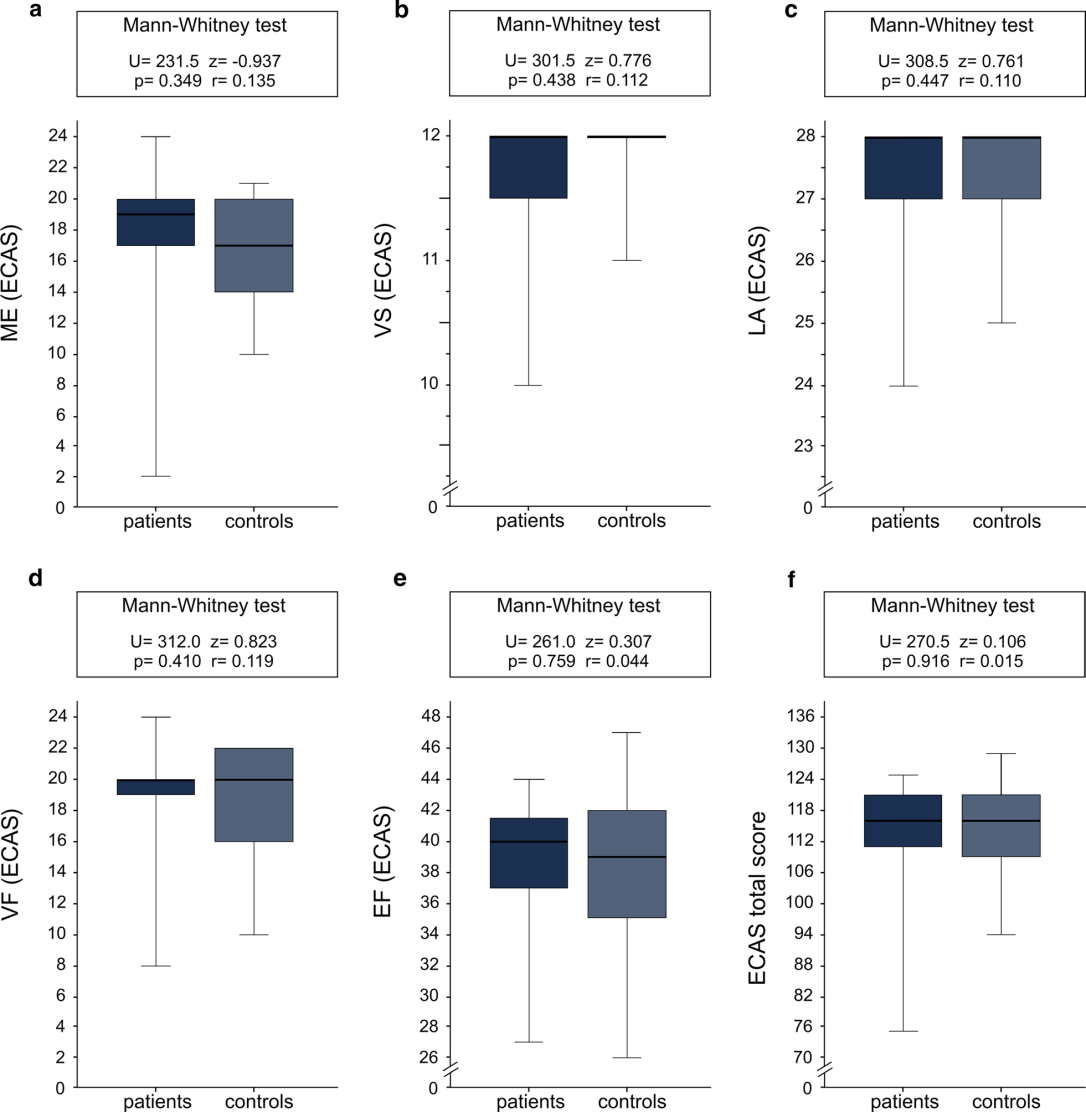
Scores of adult patients with SMA (N = 29) and controls (N = 19) in the cognitive domains of ECAS. ECAS = Edinburgh Cognitive and Behavioural ALS (amyotrophic lateral sclerosis) Screen with the following domains (maximum scores are indicated in brackets): ME = memory (max. 24); VS = visuospatial abilities (max. 12); LA = language (max. 28); VF = verbal fluency (max. 24); EF = executive function (max. 48); the ECAS total score has a maximum of 136 points

Except for the ECAS total score, none of the cognitive domains of ECAS showed a significant association with sex, age or years of education (correlation of age and ECAS total score: τ = 0.30, *p* = 0.023; Fig. 1). Executive function did not significantly differ between patients with type II (median = 40.50) and type III SMA (median = 38.00) (U = 66.50, z = − 1.654, *p* = 0.101, r = 0.31). This was also the case for the other cognitive domains and the ECAS total score. In those N = 25 patients, for whom both motor function and ECAS were evaluated, a significant inverse correlation between motor function and executive function was found (τ = − 0.36, *p* = 0.018; Fig. 2). Lower motor function was associated with better executive function. None of the other domains of ECAS showed a significant association with motor function. No significant association of disease onset with executive function was found (τ = − 0.14, *p* = 0.358), nor with any other domain of ECAS. In contrast, a highly significant correlation between disease onset and motor function was seen (τ = 0.69, *p* < 0.001; Fig. 3). Those with an earlier disease onset exhibited significantly lower physical function at the time of the study. Adults with SMA and neurologically healthy controls did not differ in any of the domains of the ECAS (Fig. 4).

**Fig. 5:**
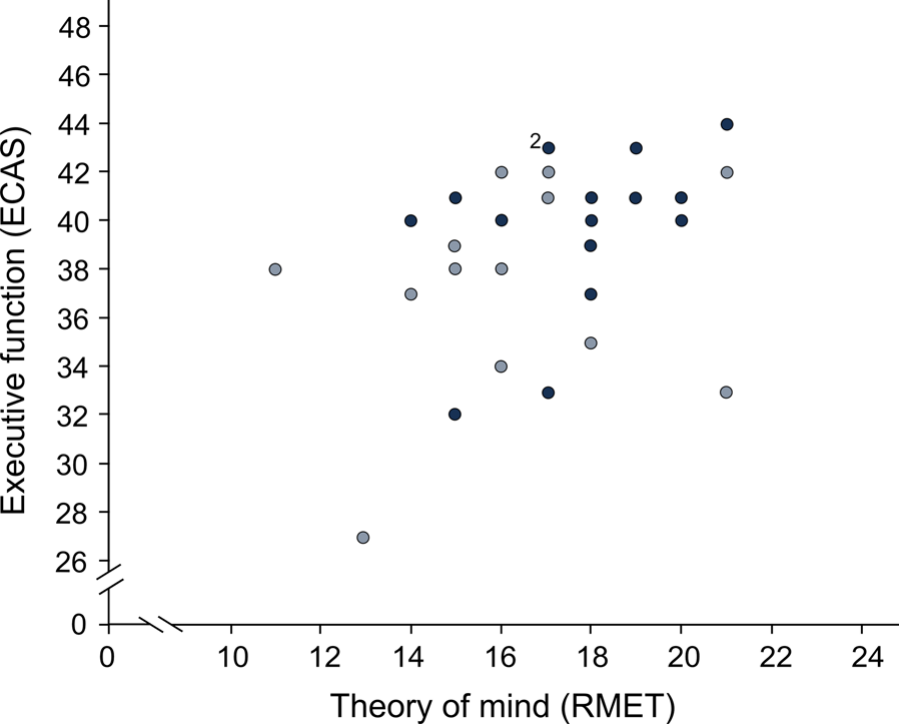
Association between executive function (ECAS domain) and theory of mind (RMET; as a measure of social cognition) in adult patients with SMA (N = 29). Dark blue = SMA type II, light blue = SMA type III; ECAS = Edinburgh Cognitive and Behavioural ALS (amyotrophic lateral sclerosis) Screen, domain for “executive function” (score 0 to 48); RMET = Reading the Mind in the Eyes Test, a test for theory of mind with scores between 0 and 24; the data point labelled “2”, represents two patients; the data show a significant correlation of executive function in ECAS and theory of mind as a measure of social cognition in RMET (τ = 0.30, p = 0.034)

**Fig. 6:**
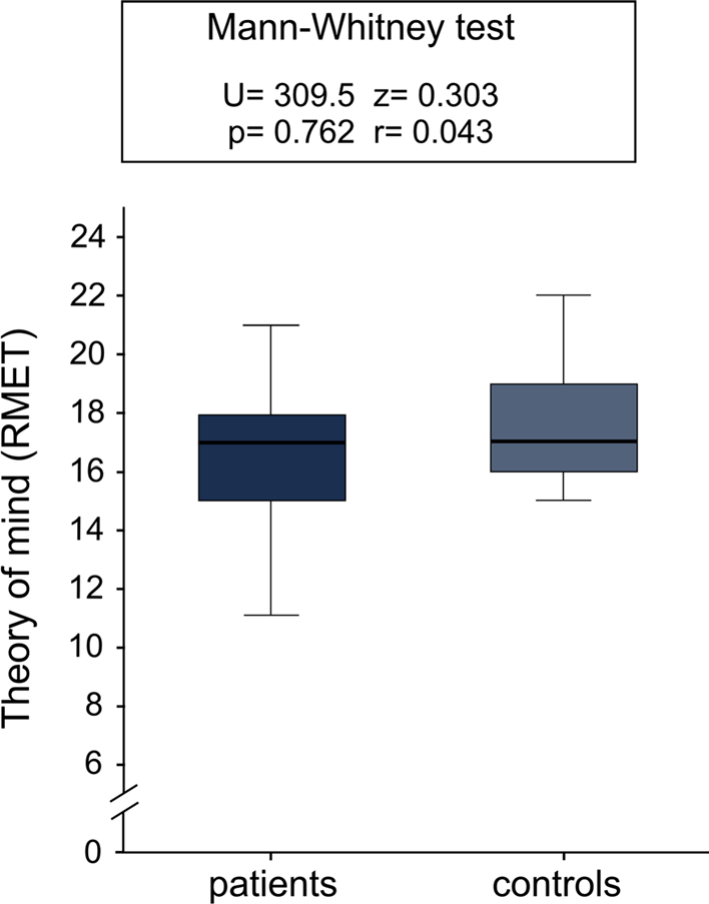
Scores of adult patients with SMA (N = 31) and controls (N = 19) in the RMET for theory of mind as a measure of social cognition. RMET = Reading the Mind in the Eyes Test, a test for theory of mind with scores between 0 and 24; patient group and control group do not differ significantly in their expertise in theory of mind

Performance in RMET—as a measure of social cognition—was not associated with sex, age or years of education. However, performance in RMET was significantly correlated with executive function (measured with ECAS) (τ = 0.30, *p* = 0.034; Fig. 5). RMET scores did not show any significant difference between type II and type III SMA, but a trend towards a better performance in type II SMA was noticed (t(25.187) = 1.882, *p* = 0.071, d = 0.68). Moreover, a better performance in RMET was slightly but not significantly associated with lower motor function (τ = − 0.25, p = 0.091). There was no significant correlation of RMET scores and age at disease onset (τ = − 0.24, *p* = 0.106). Besides, we did not capture any difference of RMET results of patients compared to controls (Fig. 6).

## Discussion

The study shows that cognitive abilities of adult SMA patients are comparable to those of healthy individuals. This implies that cognitive development remains unimpaired beyond adolescence and that cognitive abilities acquired in childhood and adolescence are preserved in adulthood, unaffected by further progression of the disease. However, cognitive abilities superior to neurologically healthy peers, as they have been found in adolescents, could not be demonstrated. Studies on children and adolescents suggested a normal (or even enhanced) development of cognitive abilities despite SMN protein shortage and the developmental role of this protein in the brain [9–18]. This work provides first evidence that, even in adulthood, cognitive abilities are not affected (to a clinically apparent extent). However, it has to be noted that individual SMN protein levels of patients were not evaluated in this study, but are a general feature of SMA. It was found that among patients, those with a lower motor function exhibited better executive function. Since motor function was significantly associated with disease onset, those with the lowest motor function are also those who have suffered from the most dramatic impairment of physical function early on in their development. Still, this subgroup of patients exhibited the best executive functions. This is striking because physical interaction with the environment is known to be an important factor for cognitive development [6]. The inverse correlation of executive and physical function may be explained by the, already mentioned, significant correlation of disease onset with motor function. Patients with a particularly early disease onset might have an earlier—and therefore more effective—cognitive adaptation to their physical disability, where they reallocate cognitive resources no longer needed for motor tasks [19] or benefit from interactional mechanisms with their caregivers [14]. However, this postulated adaptation apparently does not overcompensate above the level of executive functioning found in healthy individuals. The fact that this association of cognitive function with physical function could not be found for any other cognitive domain, raises the question if executive functions are especially important for patients with SMA. It seems plausible that problem-solving, flexible thinking and adaptation to new circumstances, which are all characteristics of executive function, are relevant to adapting to a life with a progressive neuromuscular disease. Thus, executive functions might play a special role for patients with SMA and coping with physical restrictions in general. What poses an explanatory difficulty is the fact that disease onset itself did not correlate with executive function. A possible explanation is that the relevant trigger for cognitive adaptation might not be an early disease onset in itself but motor impairment at early stages of life. Due to the variability of the disease course, the association of disease onset and early severe motor impairment is imperfect. Interestingly, social cognition (which shares many functional substrates with executive function and is thus closely associated with executive function) was not superior in SMA compared to controls, contrary to what was hypothesised based on Dubowitz’ observations [10]. In accordance with executive function, there was a trend of lower physical function being associated with better social cognition and type II SMA performing better than type III SMA patients. This fact hints at the possible role of interactional mechanisms in cognitive adaptation in the course of SMA [14], but this remains speculative and needs further investigation in future studies.

A major limitation of the study is the fact that it follows a cross-sectional design with no evidence for causal interactions of cognitive profile and SMN pathology per se, SMN protein level or nusinersen treatment. What we report here is descriptive data on cognitive performance, early on in the course of nusinersen treatment, without any expected effect of nusinersen treatment on outcome measures (which is why we also included N = 4 without treatment). Since mere correlation does not imply causation, any causal associations remain purely speculative.

A further limitation of the study is the fact that this cohort did not feature any patients with type I SMA and exhibited a relatively high proportion of patients with type III SMA. This is owed to the focus on adult patients and the natural course of the disease with type III SMA patients reaching adulthood in most cases and type I SMA reaching adulthood only in rare cases [3]. Thus, our findings may apply to adult SMA types II and III patients only and cannot be generalized and applied to (the rare) cases of adult SMA type I.

Further, there is an imbalance of the sexes in the study cohort with 65% male patients. Although sex had no significant influence on the target constructs of this study, a selection bias introduced by this random error cannot be ruled out completely. Furthermore, the sex distribution in the patient group and the control group differed considerably. This weakness in the matching can be justified again by the fact that sex had no influence on the target constructs of this study.

Finally, we included a relatively small sample of patients. Nevertheless, according to our power analysis, the study had enough power to detect relevant differences between patients and control group.

## Conclusions

There seem to be some hurdles in the cognitive development of SMA patients (e.g. low SMN protein levels, restricted physical interaction with the environment, challenges in school or at university). However, patients with SMA acquire normal cognitive abilities. Lower physical function, traditionally viewed as detrimental to cognitive development, might in fact promote an enhanced cognitive adaptation to SMA. This possible adaptation is mirrored in better-developed executive functions in patients with lower physical function, while other cognitive domains seem unaffected. This suggests that executive functions might play a role for compensating for (or coping with) physical restrictions in patients with SMA.

While this explanation is only one of many possible explanations for the inverse correlation of executive function and physical function, it can be stated that restricted motor function is not associated with impaired cognitive function, neither in young age [12–18] nor in adulthood, as suggested by the hereby presented data. This knowledge will be of special importance in the future, due to the recent emergence of new therapeutic options for patients with SMA like nusinersen, risdiplam or Zolgensma^®^. With these new therapies, it can be expected that patients with SMA will get older and that the population of adults with SMA will grow in numbers and clinical relevance. Hence, it is relevant to also describe cognitive phenotypes in adults with SMA, who will increase in numbers due to therapeutically expanded life expectancy.

## Methods

### Subjects

A total of N = 31 adult SMA patients (age range 19.1–64.6 years, median age: 35.9 years; Table 1) were consecutively included between June 2017 and November 2018. N = 27 of them were enrolled at the inpatient clinics of Hannover Medical School (MHH) (N = 16) and of Ulm University (N = 11). These patients were recruited in the context of their treatment with nusinersen. The remaining four patients were enrolled and assessed at the outpatient clinic of Ulm University and did not receive any treatment with nusinersen. Assessment of patients was carried out early in the two-month loading-phase of treatment, within 2 weeks after the start of therapy with nusinersen. Measurement of motor function was conducted using the Hammersmith Functional Motor Scale Expanded (HFMSE) [23] for those N = 27 patients who were treated in an inpatient setting. The cohort is characterized in Table 1.

**Table 1:** Sociodemographic and disease-specific characterisation of patient and control group

	Patient group	Control group
N	31	19
Age (years)		
Max	64.6	62.5
u. quart	46.8	49.6
Median	35.9	36.2
l. quart	25.0	24.6
min	19.1	21.5
Sex		
Male	20 (64.5%)	10 (52.6%)
Female	11 (35.5%)	9 (47.4%)
Education (years)		
Max	26	23
u. quart	18	18
Median	16	16
l. quart	14	13
Min	9	10
SMA type		
I	0 (0.0%)	
II	16 (51.6%)	
III	15 (48.4%)	
Motor function (HFMSE)		
N	27	
Max	66	
u. quart	42	
Median	4	
l. quart	0	
Min	0	

HFMSE = Hammersmith Functional Motor Scale Expanded, a test for physical function with scores ranging from 0 (worst) to 66 (best); u. quart. = upper quartile; l. quart. = lower quartile; interqu. = interquartile range

For comparison, a control group of N = 19 neurologically healthy individuals matched to patients for age, sex and years of education was established. Controls were all either clinically healthy or suffered from orthopaedic diseases. Exclusion criteria encompassed any neurological disease or condition associated with relevant long-term physical incapacitation. Also, none were under psychoactive drug medication. Controls were either consecutively recruited from the orthopaedic inpatient clinic at Ulm University Clinic or from the personal surroundings of authors.

### Neuropsychology

To evaluate the general cognitive profile, language, verbal fluency, executive function, memory and visuospatial abilities were assessed with the Edinburgh Cognitive and Behavioural ALS Screen (ECAS). ECAS was originally developed as a screening tool for cognitive and behavioural alterations in patients with ALS [20]. It consists of 16 tests, subsumed under the five domains named above. Maximum group scores for ECAS are listed in Fig. 4. In the past, this test has not been validated for patients with SMA. Nevertheless, ECAS seems an appropriate tool for this collective of patients, since the test was specifically designed for patients with a neurodegenerative disease with physical handicap.

Further, ToM as a measure of social cognition, was assessed with the Reading the Mind in the Eyes Test (RMET). RMET is a test developed to detect social cognition [21]. A German modified version with 24 (instead of 36) items was used [22]. Patients were shown 24 black-and-white photographs of pairs of eyes. For each pair of eyes, the correct emotion expressed by them had to be chosen from four response options. The score in RMET equals the number of correct answers (max. 24; higher scores responding to higher social cognition).

While all N = 31 patients completed the RMET, only N = 29 patients completed the ECAS.

### Statistics

Analyses were conducted using IBM SPSS Statistics 25. After testing for normality with the Shapiro-Wilks test, independent subgroups were compared with either the Mann–Whitney test or the t-test. Correlations were carried out with Kendall’s tau. The threshold for significance was set with *p* ≤ 0.05. An a priori power analysis was conducted using G*Power 3 to determine the required sample size to detect superiority of cognitive function in patients compared to controls (measured with ECAS). We used a one-tailed test, a medium estimated effect size (d = 0.77), an alpha of 0.05 and an allocation ratio of 1.5. Results showed that for a power of 0.80, a sample of 29 patients and 19 controls was required. The estimated effect size was based on the work of Gontard et al. [18], who found adolescents (12–18 years) with SMA to have superior cognitive abilities compared to healthy peers (d = 0.73). For adult SMA patients, we expected an even higher effect size (d = 0.77).

## Data Availability

The datasets used and/or analyses during the current study are available from the corresponding author on reasonable request.
